# Crystal structures of murine angiogenin-2 and -3 – probing ‘structure – function’ relationships amongst angiogenin homologues

**DOI:** 10.1111/febs.12071

**Published:** 2012-12-11

**Authors:** Shalini Iyer, Daniel E Holloway, K Ravi Acharya

**Affiliations:** Department of Biology and Biochemistry, University of BathUK

**Keywords:** angiogenin, crystal structure, enzyme mechanism, metal-ion binding, ribonuclease A

## Abstract

Angiogenin (Ang) is a potent inducer of neovascularization. Point mutations in human Ang have been linked to cancer progression and two neurodegenerative diseases: amyotrophic lateral sclerosis and Parkinson's disease. Intensive structural and functional analyses of Ang have been paramount in assigning functions to this novel homologue of bovine pancreatic RNase A. However, inhibitor-binding studies with crystalline Ang (for designing potential anti-cancer drugs) have been hampered as a result of the inaccessibility of the active site. Experiments with the murine homologues of Ang have not only overcome the obvious practical limitations encountered when studying the role of a human protein in healthy individuals, but also the crystal structures of murine angiogenins (mAng and mAng-4) have revealed themselves to have greater potential for the visualization of small-molecule inhibitor binding at the active site. In the present study, we report the crystal structures of two more murine Ang paralogues, mAng-2 and mAng-3, at 1.6 and 1.8 Å resolution, respectively. These constitute the first crystal structures of an Ang with a zinc ion bound at the active site and provide some insight into the possible mode of inhibition of the ribonucleolytic activity of the enzyme by these divalent cations. Both structures show that the residues forming the putative P_1_, B_1_ and B_2_ subsites occupy positions similar to their counterparts in human Ang and are likely to have conserved roles. However, a less obtrusive conformation of the C-terminal segment in mAng-3 and the presence of a sulfate ion in the B_1_ subsite of mAng-2 suggest that these proteins have the potential to be used for inhibitor-binding studies. We also discuss the biological relevance of the structural similarities and differences between the different Ang homologues.

**Database:**

The atomic coordinates and structure factors for mAng-2 (3ZBV) and mAng-3 (3ZBW) have been deposited in the Protein Data Bank, Research Collaboratory for Structural Bioinformatics, Rutgers University, New Brunswick, NJ, USA (http://www.rcsb.org/).

**Structured digital abstract:**

1. mAng2 and mAng3 bind by x-ray crystallography (View interaction)

## Introduction

The bovine pancreatic RNase A superfamily comprises a group of homologous proteins that has been intensely targeted in biochemical, structural, enzymatic and evolutionary studies. Angiogenin (Ang, a 14-kDa protein), an unusual member of the superfamily, is a potent inducer of blood vessel formation that was first isolated from the conditioned medium of cultured HT-29 human colon adenocarcinoma cells based solely on its angiogenic activity 1. The overall structure of human Ang (hAng), as determined by Acharya *et al*. 2, not only showed it to have a structural fold similar to that of RNase A 3, but also revealed it to be markedly different in several distinct areas. It can stimulate angiogenesis and, although the ribonucleolytic activity of the protein is rather weak, it is critical for its angiogenic properties 4,5. Studies with Ang antagonists in athymic mice pointed to a critical role played by this protein in the formation of some human tumours 6–9 and its increased expression has been correlated with diverse cancers in several clinical studies. The *ang* gene was the first loss-of-function gene identified in amyotrophic lateral sclerosis (ALS), a progressive neurodegenerative disease characterized by the selective destruction of motor neurons 10–13. More recently, *ang* has also been shown to be implicated in Parkinson's disease 13. The different mutations identified and characterized so far have all proven to be loss-of-function mutations 14–16. A detailed structure–function study by Thiyagarajan *et al*. 2012 on several of these Ang-ALS variants provided the first insights into the cellular and molecular mechanisms underlying their role in ALS. Ang, however, has a nonpathological function to play as well, being a constituent of normal plasma 18 and a secreted product of a wide variety of normal cells 19,20. Indeed, endogenous Ang is a general requirement for cell proliferation and angiogenesis 21.

It has been observed that four different aspects of Ang are required for its angiogenicity: (a) ribonucleolytic activity; (b) stimulation of basement membrane degradation; (c) stimulation of signal transduction; and (d) nuclear translocation. Both the enzymatic and angiogenic activities of hAng are inhibited by its interaction with a 50-kDa leucine-rich repeat protein, human placental RNase inhibitor (hRI) (*K*_i_ < 1 fm) 22. The crystal structure of hRI–hAng 23, in conjunction with mutagenesis studies 24–26, provided insight into the residues involved from both hAng and hRI with respect to forming this extremely tight association. Despite a wealth of information already gathered for the role of human Ang, there are practical limitations in studying the role of the human protein. The murine Ang system, on the other hand, offers an alternative route for studying the physiological and developmental roles of Ang in greater depth.

**Fig 1:**
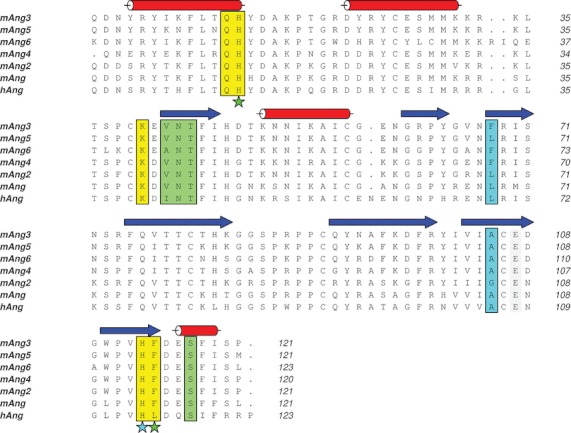
Sequence alignment of the human and murine angiogenins. Elements of secondary structure are indicated by red cylinders (α-helices) and blue arrows (β-strands). Secondary structures for mAng-5 and mAng-6 are predicted based on the known Ang structures. Residues that form the putative substrate-binding subsites are highlighted: P_1_ subsite residues are shaded in yellow; B_1_ subsite residues are shaded in green and B_2_ subsite residues are shaded in cyan. The stars highlight those active site residues that belong to two subsites and are coloured according to the additional subsite. The sequences were aligned using clustalw 74. Features were added to the alignment using aline 75.

The murine Ang family, over the course of evolution, has expanded to accommodate six members: mAng (the murine orthologue of hAng), mAng-2 (also known as mAngRP or mAng-related protein), mAng-3, mAng-4, mAng-5 and mAng-6 27–31 (Fig. 1). Evolutionary studies by Codoñer *et al*. 2010 suggest that there is functional divergence amongst the mAng paralogues even though the 3D models for the proteins are extremely similar. Experiments have shown that mAng, mAng-3 and mAng-4 have angiogenic activities comparable to that of hAng, whereas mAng-2 is not angiogenic 33–35. It has also been shown mAng-4 functions as a Paneth cell-derived antimicrobial peptide important in epithelial host defence in the small intestine 36. mAng-4 is also expressed by the goblet cells of the large intestine after bacterial infection and this expression is controlled by interleukin-3 37. At present, only the crystal structures of mAng and mAng-4 35,38 are known, and the topologies of the two murine enzymes are highly similar to those of hAng and RNase A 2,3. Previously, we presented in-depth analysis of the structure–function relationships of mAng and mAng-4 by means of site-directed mutagenesis 35,38. In the present study, we present the X-ray crystal structures of mAng-2 and mAng-3. We have examined the similarities and differences that these two structures have with the structures of the other two murine paralogues, as well as that of human Ang. Insights gained through comparative analyses of the available structural and functional data provide a means for probing the structure–function relationships amongst the different Ang homologues.

## Results

### Ribonucleolytic activity

**Fig 2:**
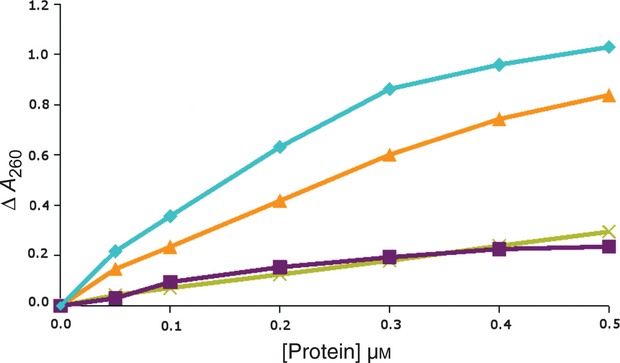
Comparative tRNA cleavage activities of murine and human angiogenins. Ribonucleolytic assays measuring the release of perchloric acid-soluble fragments catalysed by mAng-2 (orange), mAng-3 (olive green), mAng (pink) and hAng (cyan) as described previously 65,66. Each data point represents the mean of at least two experiments. Human angiogenin was used as a reference sample. Assays were conducted for 2 h at 37 °C in reaction buffer consisting of 33 mm Na-HEPES, pH 7.0, 33 mm NaCl, 2 mg·mL^−1^ tRNA and 0.1 mg·mL^−1^ BSA, plus the indicated concentrations of recombinant protein.

Untagged recombinant proteins were employed throughout the present study. mAng-2 differed from mAng and mAng-3 in that the Pca1 (N-terminal pyroglutamate residue) was replaced by Met(-1)-Gln1. It has been shown previously that such a replacement does not affect the ribonucleolytic activity of hAng owing to the presence of a long N-terminal segment that positions Pca1 far from the active site 33. Similarly, it is expected that the replacement will not affect the ribonucleolytic activity of the murine Angs assayed in the present study. Mouse Ang-3 is similar to mAng, with an activity several fold lower than that of hAng, whereas mAng-2 is almost 80% active compared to the human form of the protein (Fig. 2).

#### Structural overview

**Fig 3:**
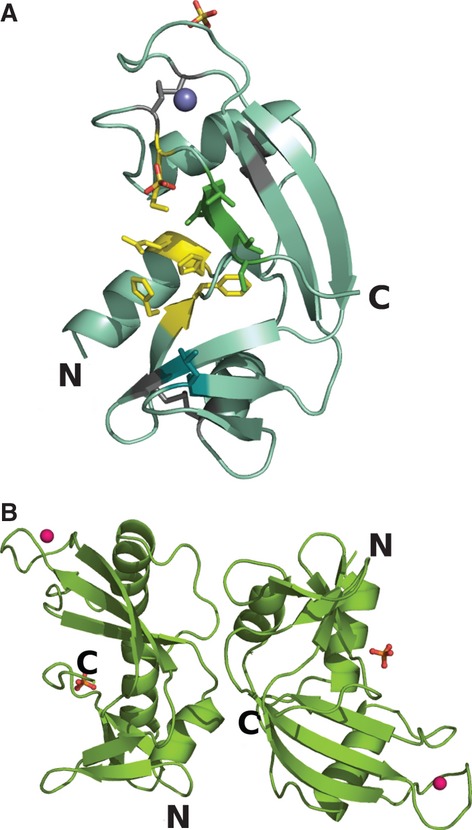
Crystal structures of mAng-2 (A) and mAng-3 (B). mAng-2 is coloured greencyan and mAng-3 is shown in splitpea green. The 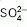 ions in both structures are represented as a ball-and-stick and coloured according to their atoms (sulfur atoms in yellow and the oxygen atoms in red). The Zn^2+^ ions are represented as spheres in slate blue (mAng-2) and magenta (mAng-3). The N- and C-terminal ends of all monomers have been labelled. In (A) residues that form the putative substrate-binding subsites are shown in stick form: P_1_ subsite residues are coloured yellow; B_1_ subsite residues are coloured green and B_2_ subsite residues are coloured cyan.

**Fig 4:**
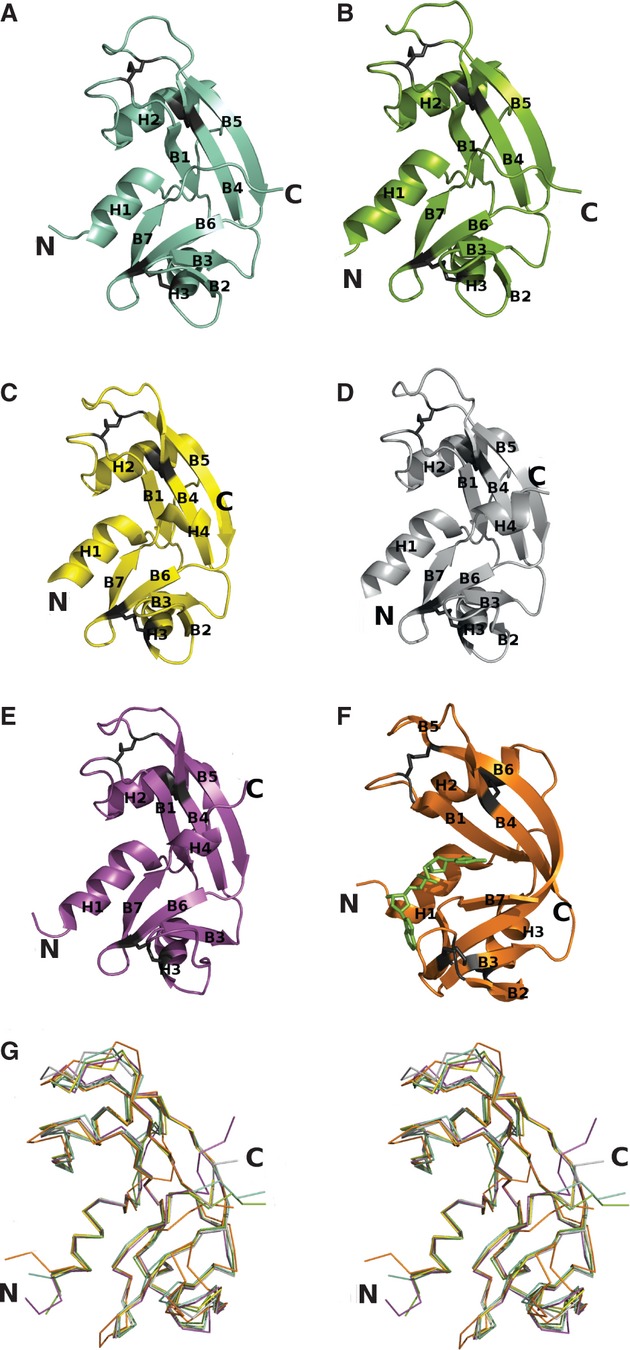
Topological comparison of Ang homologues. Ribbon diagrams of (A) mAng-2, (B) mAng-3, (C) mAng, (D) mAng-4, (E) hAng (PDB code: 1B1I) 49 and (F) RNase A (PDB code: 1RUV) 76. Elements of secondary structure are labelled, as are the N- and C-terminal extremities of each structure. Disulfide bonds are shown in black in ball-and-stick form. Also modelled in stick form and coloured green in panel (F) is the uridine vanadate (UVC) molecule. (G) Stereo superposition of the C^α^ traces of all the six proteins. Colours correspond to those used in (A)–(F).

The crystal structures of mAng-2 and mAng-3 were determined at 1.64 and 1.80 Å resolution, respectively. Crystals for mAng-2 and mAng-3 contained one and two molecules (A and B) in the asymmetric unit, respectively. The final structures comprise residues 4–119 for both chains of mAng-2, whereas mAng-3 chains A and B comprise residues 2–121 and 1–121, respectively. Main-chain electron density for the residues listed above is well ordered. Ramachandran plots 1963 indicate that the proportions of non-proline/glycine residues in the most favoured regions are 85.3% and 88% for mAng-2 and mAng-3, respectively, with the remainder of the residues falling in the additional allowed regions of the plot for both the structures. The crystallographic statistics for both structures are provided in Table 1. The main-chain of both mAng-2 and mAng-3 adopts the familiar α/β fold that is characteristic of other Angs and members of the RNase A family (Figs 3 and 4). Both mAng-2 and mAng-3 have two deleted residues, similar to mAng, compared to the sequence of the human enzyme (Fig. 1). There is, however, a close correspondence of the core secondary structure elements when other Angs and RNase A are structurally aligned with mAng-2 and mAng-3 (Fig. 4).

**Table 1:** Crystallographic statistics

	mAng-2	mAng-3
Data collection		
X-ray wavelength (Å)	0.87	0.97
Space group	*I4*_*1*_*22*	*P*2_1_
Cell dimensions (Å)	a = b = 89.72; c = 64.55	a = 28.2; b = 95.67; c = 41.66 β = 100.99°
Resolution (Å)	40–1.64	40–1.8
Number of reflections measured	66 711	55 483
Number of unique reflections	14 855	15 155
*R*_sym_ (outermost shell)^1^ (%)^2^	6.7 (24.5)	6.2 (39.5)
*I*/σ*I* (outermost shell)	16.9 (5.2)	15.9 (2.0)
Completeness (outermost shell) (%)	97.0 (99.0)	75.4 (23.2)
Refinement		
*R*_cryst_ (%)^3^	19.8	16.5
*R*_free_ (%)^4^	20.3	22.6
Number of atoms (total)	1118	2207
Protein atoms	962	1969
Water	139	222
Ions		
Zn^2+^	1	2
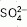	10	10
Ligand	6 (glycerol)	4 (acetate)
rmsd from ideality		
Bond length (Å)	0.008	0.008
Bond angles (°)	1.257	1.119
Mean *B*-factor (Å^2^)	19.4	18.7

a Outermost shell (Å): mAng-2, 1.70–1.64 Å; mAng-3, 1.9–1.8 Å.

b *R*_sym_ = ∑(|*I*_j_ − <*I*>|)/∑<*I*> where *I*_*j*_ is the observed intensity of reflection *j*, and <*I*> is the mean intensity of multiple observations.

c *R*_cryst_ = ∑||*F*_o_| − |*F*_c_||/∑|*F*_o_|, where *F*_o_ and *F*_c_ are the observed and calculated structure factor amplitudes, respectively.

d *R*_free_ is equal to *R*_cryst_ for a randomly selected 5% reflections not used in the refinement.

### Dimer interface

The crystal structure of mAng-3 contains a dimer in the asymmetric unit (Fig. 3). The two protomers of mAng-3 superpose with an rmsd of 0.3 Å. The interface of mAng-3 buries ∼450 Å^2^ of solvent-accessible surface as calculated using the pisa interface server (http://www.ebi.ac.uk/msd-srv/prot_int/pistart.html). The mAng-3 dimer interface has 16 residues per subunit at the interface and, although the residues located at the contact surface include a number of polar and charged side-chains, no ionic interactions and only two hydrogen-bonding contacts are observed.

The pisa server suggests that the dimerization of mAng-3 is not constitutive. The protein–protein interactions are nonspecific and of the same type as those that stabilize specific complexes such as those between a ligand and its receptor or between an antigen and its antibody. They are distributed among several small patches and exhibit poor shape complementarity. Based on the molecular contacts at the interface, pisa calculates a complexation significance score (CSS) that, on a scale from 0 to 1, indicates the probability that a packing interface generated through crystallization might represent a significant feature in solution. The CSS for the dimer interface of mAng-3 is 0, suggesting that dimerization is merely a result of crystal packing.

Interestingly, analysis of the symmetry-related molecules in the mAng-2 crystal structure revealed a two-fold crystallographic dimer that is particularly intimate. The buried surface area at the interface between the two molecules is ∼1100 Å^2^, consisting of 33 amino acid residues from each molecule with a total of ten ionic interactions across the interface. Even though pisa calculated a CSS of 0.06 for the mAng-2 crystallographic dimer, the buried interface at the mAng-2 dimer is of considerable size and contains approximately one polar interaction per 150 Å^2^, a high figure 40.

### Interactions mediated by Zn^2+^ and 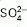 ions

**Fig 5:**
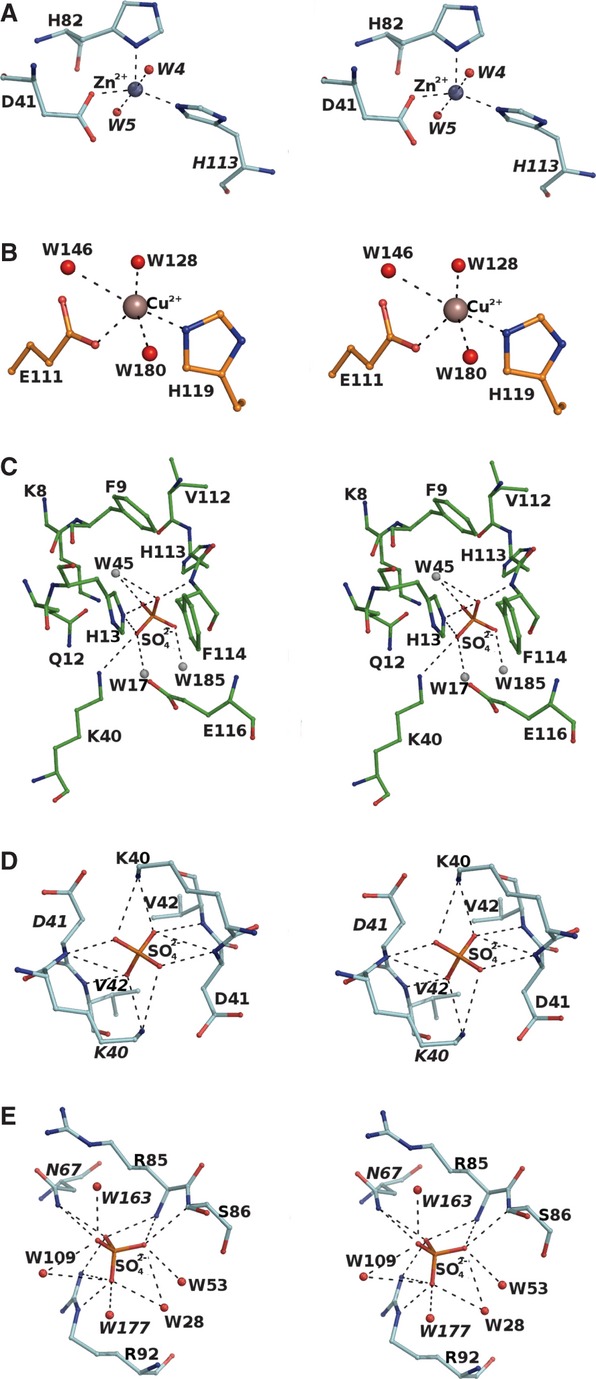
Ion-mediated interactions. (A) Stereoview of the Zn^2+^-binding site in mAng-2. (B) Stereoview of the Cu^2+^-binding site in RNase A–Cu^2+^ complex (PDB code: 1AQP) 41. (C) Stereoview of the 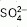 binding site in mAng-3. (D) Stereoview of the central 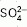-binding site in mAng-2. (E) Stereoview of the peripheral 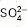-binding site in mAng-2. The interacting residues are coloured according to their elements: carbon (cyan in mAng-2; green in mAng-3 and orange in RNase A; nitrogen, blue; oxygen, red). The water molecules are shown as grey or red spheres, whereas the Zn^2+^ ion is shown in magenta. The dashed lines represent potential hydrogen bonds. mAng-2 residues contributed by the symmetry-related protomer in (A), (D) and (E) are italicized.

The crystal structures of both mAng-2 and mAng-3 contain Zn^2+^ and 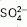 ions. The mAng-2 structure has a total of one Zn^2+^ ion and two 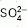 ions incorporated into the asymmetric unit by virtue of the crystallization solution, whereas the mAng-3 structure has two Zn^2+^ ions and two 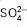 ions. Interestingly, in the mAng-2 structure, His82, Asp41 and two water molecules from the protomer in the asymmetric unit, along with His113 (an active site residue) from a symmetry-related mate coordinate the Zn^2+^ ion to complete a penta-coordination (Fig. 5A). A reciprocal set of interactions makes up the symmetry-related Zn^2+^ binding site (Table 2), resulting in a head-to-tail packing. The two Zn^2+^ binding sites in the mAng-3 structure are completely different from each other (Table 2). One of the zinc ions has only two interactions: one with the hydroxyl group of Tyr14 and the other with a water molecule. The second zinc site has five interactions: one with each of His82 and Glu41, two with water molecules and one with an acetate ion. Although it contains no true coordination bonds (all the bond lengths are > 2.3 Å), the second zinc-binding site in mAng-3 is similar to the two zinc-binding sites in the mAng-2 dimer.

**Table 2:** Zinc ion binding in the active site of mAng-2 and mAng-3. SYM, symmetry related molecule

mAng-2			mAng-3		
Source	Target	Distance (Å)	Source	Target	Distance (Å)
Zn	His 113B NE2 (SYM)	2.0	Zn 1	Tyr 14A OH	2.8
	Water (SYM)	2.2		Water	2.7
	His 82A ND1	2.1		–	–
	Water (SYM)	2.2		–	–
	Asp 41A OD1	2.0		–	–
Zn (SYM)	His 113A NE2	2.0	Zn 2	Acetate OXT	2.6
	Water	2.2		Water	2.7
	His 82B ND1 (SYM)	2.1		His 82A ND1	2.3
	Water	2.2		Water	2.5
	Asp 41B OD1 (SYM)	2.0		Glu 41A OE1	2.6

The zinc-binding site in mAng-2 resembles one of the copper-binding sites in the RNase A–Cu^2+^ complex (Fig. 5B) [Protein Data Bank (PDB) code: 1AQP] 41 in some aspects. The metal-binding sites share a common catalytic His residue (His113 in mAng-2 and His119 in RNase A). Another similarity between the metal-binding sites in mAng-2 and RNase A is that the ions bind a side-chain carboxyl group (donated by Asp41 and Glu111), respectively. In the RNase A–Cu^2+^ complex structure, three water molecules make up the rest of the coordination bonds at the metal-binding site, whereas two water molecules and the ND1 atom of His82 provide the remaining coordination groups for the zinc ion in mAng-2.

The sulfate-binding sites (Table 3) in mAng-2 and mAng-3 are quite different from one another. In the mAng-3 structure, the binding site architecture is very similar to that observed for phosphate and sulfate ions in other Ang structures and both anions ligate the same five P_1_ subsite residues: Gln12, His13, Lys40, His113 and Phe114 (Fig. 5C). However, in the mAng-2 structure, the central sulfate ion interacts with none of the above P_1_ subsite residues except for the catalytic lysine (Lys40) (Fig. 5D). The presence of a Zn^2+^ ion at the active site forces the side-chain of Lys40 to orient itself away from the catalytic site. As a result, this ion interacts with the main-chain nitrogen and oxygen atoms of Asp41 and Val42 from opposing symmetry-mates. The binding site of the other sulfate ion in the mAng-2 structure bears no resemblance to any interactions observed within previously elucidated structures of angiogenins. The anion is well coordinated, making hydrogen bonds to Asn67, Arg85, Ser86 and Arg92 (Fig. 5E). The crystallographic mAng-2 dimer presents a symmetry-related sulfate ion that is located directly opposite to the above mentioned sulfate ion at the other end of the dimer and is equally populated.

**Table 3:** Potential hydrogen bonds (identified using hbplus 77) between protein and anionic ligands (PO_4_ and/or SO_4_) in the mAng-2, mAng-3, mAng-1 and hAng complexes. The upper limit for the donor-acceptor distance was 3.3 Å; the lower limit for the donor-hydrogen-acceptor angle is 105°. Bond angles are not given where the hydrogen position is ambiguous

Ligand acceptor atom	mAng-2–SO_4_	mAng-3–SO_4_	mAng–PO_4_ (PDB code: 2BWL)	mAng–SO_4_ (PDB code: 2BWK)	hAng–PO_4_ (PDB code: 1HBY)
O1	B Lys40 NZ (2.8; 144.5) B Val42 N (3.0; 150.5) B Asp41 N (3.1)^a^	B Phe114 O (2.8)^a^ Water (2.6)	His13 NE2 (2.8; 139) Phe114 N (3.0; 166)	Phe114 N (2.8; 166)	His113 NE2 (3.2; 136) Leu115 N (3.0; 169)
O2	A Lys40 NZ (2.9; 163.2) A Val42 N (2.9; 147.5) A Asp41 N (3.1)^a^	B Gln12 OE1 (3.1)^a^ B His13 NE2 (2.7)^a^ B Lys40 NZ (2.9; 133) Water (2.8)	Gln12 NE2A(2.9; 117) Lys40 NZ (2.9; 135)	Gln12 NE2B (2.8; 112) His13 NE2 (3.0; 160) Lys40 NZ (3.1; 130)	Gln12 NE2 (2.6; 119) His13 NE2 (3.2; 158) Lys40 NZ (3.2; 105)
O3	A Lys40 NZ (3.3)^a^ B Asp41 N (2.8)^a^	B His13 NE2 (2.9)^a^ B His113 ND1A (3.2)^a^ B Phe114 N (2.8; 165) Water (3.0)	His113 ND1A (2.9;153)^b^ His113 ND1B (3.2; 148)^b^ His113 ND1A (3.1; 153)^c^	His113 ND1B (2.9; 158)	His114 ND1 (3.4; 146)
O4	A Asp41 N (3.0; 173.1)^a^ B Lys40 NZ (3.3)^a^	B His113 ND1A (2.9; 132) B His113 ND1B (3.4)^a^	–	–	–
O1		Water (2.4)			
O2		A His13 NE2 (3.1)^a^ A Phe114 N (2.8; 158) Water (2.8)			
O3		A Gln12 NE2 (3.3)^a^ Water (2.9)^a^			
O4		A Gln12 NE2 (3.2)^a^ A His13 NE2 (2.9; 160) A Lys40 NZ (3.0; 137) Water (3.0); Water (3.4)^a^			
O1	B Asn67 ND2 (3.4)^a^ Water (3.0); Water (2.7)				
O2	B Asn67 ND2 (2.8; 163.2) A Arg85 N (3.0; 157.2) A Arg92 NH2 (2.9; 169.2)				
O3	A Ser86 N (2.8; 147) A Arg85 N (3.0)^a^ Water (3.2); Water (3.2)				
O4	A Arg92 NE (2.7; 154.5) A Arg92 NH2 (3.3)^a^ Water (2.9); Water (2.9)^a^				

a Alternative interactions identified by contact 78.

b,c Interactions with orientation A and B of phosphate ion in mAng–PO_4_ (PDB code: 2BWL) 38.

### Substrate-binding subsites

#### P_1_-subsite and the catalytic centre

The catalytic triad at the P_1_-subsite is robustly conserved in mAng-2 and mAng-3, in the form of His13, Lys40 and His113. When compared with the previously-determined structures of RNase A superfamily members, the positioning and conformation of these three residues indicates that they are involved in the catalysis of the scissile phosphodiester linkage of RNA substrates, whereas the side-chain of Gln12 and the main-chain of Phe114 are most likely engaged in other phosphate-binding interactions 42.

In RNase A homologues, the C-terminal catalytic histidine (His113) may adopt two conformations: conformation A (productive) and conformation B (nonproductive) 43. In mAng-2, the productive conformation is observed for this residue. There are no intramolecular interactions in mAng-2 that might stabilize His113 in conformation A (as is the case in the hAng structure), although this residue does interact with a zinc ion. In mAng-3, His113 from chain A is observed in conformation B, whereas the same residue takes on both productive and nonproductive conformations in the other chain. The nonproductive conformation of His113 in chain A of mAng-3 forms a hydrogen bond with an oxygen atom of the nearby sulfate ion via its ND1 atom. Its NE2 atom interacts with a symmetry-related Glu107 (the counterpart of Glu111 in RNase A) instead of a water molecule as has been observed for conformation B in some structures of RNase S 44,45. The conformation of His13 and Phe114 is the same in mAng-2 and mAng-3 as that seen in the hAng structure. The orientation of Lys40, however, differs between the two murine angiogenins. In mAng-3, the side-chain of Lys40 points towards the active site of its own protomer. In mAng-2, Lys40 is expelled and ligates a sulfate ion occupying the active site of a symmetry-related molecule.

#### B_1_-subsite and the C-terminal segment

**Fig 6:**
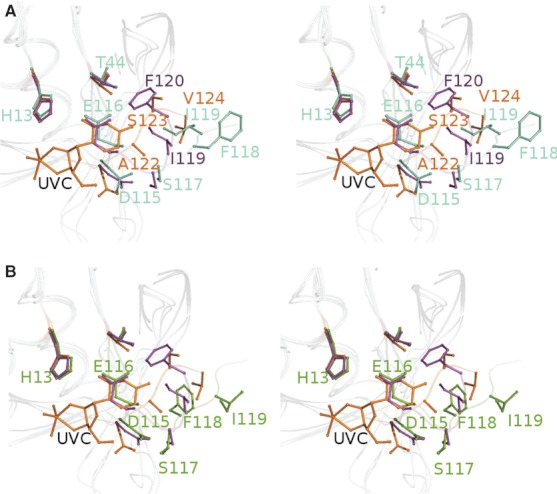
Putative B_1_ subsite and the intruding C-terminus. mAng-2, mAng-3 and hAng were aligned with the RNase A–uridine vanadate complex (PDB code: 1RUV) 76 on the basis of the C^α^ positions of His12, Lys41 and His119 in the RNase A structure. Schematic representations are shown in stereo for: (A) mAng-2 (greencyan) superposed with hAng (magenta) and RNase A (orange); and (B) mAng-3 (splitpea green) superposed with hAng (magenta) and RNase A (orange). Residues are shown in ball-and-stick form. Also modelled from the RNase A structure is the uridine vanadate (UVC) molecule. Residue labels are coloured in accordance with the structure that they represent. All the residues from mAng-2 (A) and from mAng-3 (B) are labelled. Structurally equivalent residues from hAng and RNase A have been labelled in (A).

The B_1_-subsite, which is well characterized in RNase A 46,47 binds the nucleotide base upstream of the scissile phosphodiester bond. In RNase A, the subsite is shaped as a narrow pocket and is formed by six residues of which Thr45 is the crucial component, forming primary hydrogen bonds with the polar groups of the pyrimidine bases at positions 2 and 3 47,48. The residues that are likely to form the B_1_-subsite in mAng-2 and mAng-3 are partially conserved with those that form this subsite in hAng (Fig. 1). Despite the two amino acid substitutions whereby hAng Ile42 is replaced by Val42 and hAng Leu115 is replaced by Phe114, all the residues are conventionally positioned (similar to their hAng counterparts), indicating that these residues are likely to play similar roles. The region in which the pyrimidine is predicted to bind to hAng is obstructed by the C-terminal segment (amino acids 117–123), most significantly by residues Gln117 and Phe120 2,49,50. In comparison, the active site of mAng-2 and mAng-3 is more open than that of hAng (Fig. 6). Although Glu116 of mAng-2 and mAng-3 obstructs the B_1_-subsite by making two hydrogen bonds with Thr44 that are reminiscent of those made between Gln117 and Thr44 in hAng, Ile119 in mAng-2 and mAng-3 (the spatial counterpart of Phe120 in hAng) adopts a less obstructive conformation that does not pack against the main body of the protein, more akin to the corresponding segment of RNase A (Figs 4G and 6). Instead, the C-terminal segment in the two murine angiogenins projects out and the main chain progressively deviates from this point onwards. The access route to the active site is clearer in mAng-2 and mAng-3 than in mAng and mAng-4.

#### B_2_-subsite and the H3-B2 loop

The B_2_-subsite binds the nucleotide base downstream of the scissile phosphodiester linkage. In RNase A, the residues that make up this subsite are contributed by a loop (amino acids 65–72) that is stabilized by a disulfide bridge 51. In angiogenins, however, this disulfide bond is not conserved, and the B_2_-subsite is relatively rudimentary 38,49. In mAng-2 and mAng-3, the three residues predicted to make up this subsite are Leu68 (mAng-2)/Phe68 (mAng-3), Gly105 (mAng-2)/Ala105 (mAng-3) and His113. These residues occupy positions similar to their hAng, mAng and mAng-4 counterparts and hence support the notion that any interactions they make with the substrate will be conserved.

### Cell-binding site and the nuclear localization sequence (NLS)

**Fig 7:**
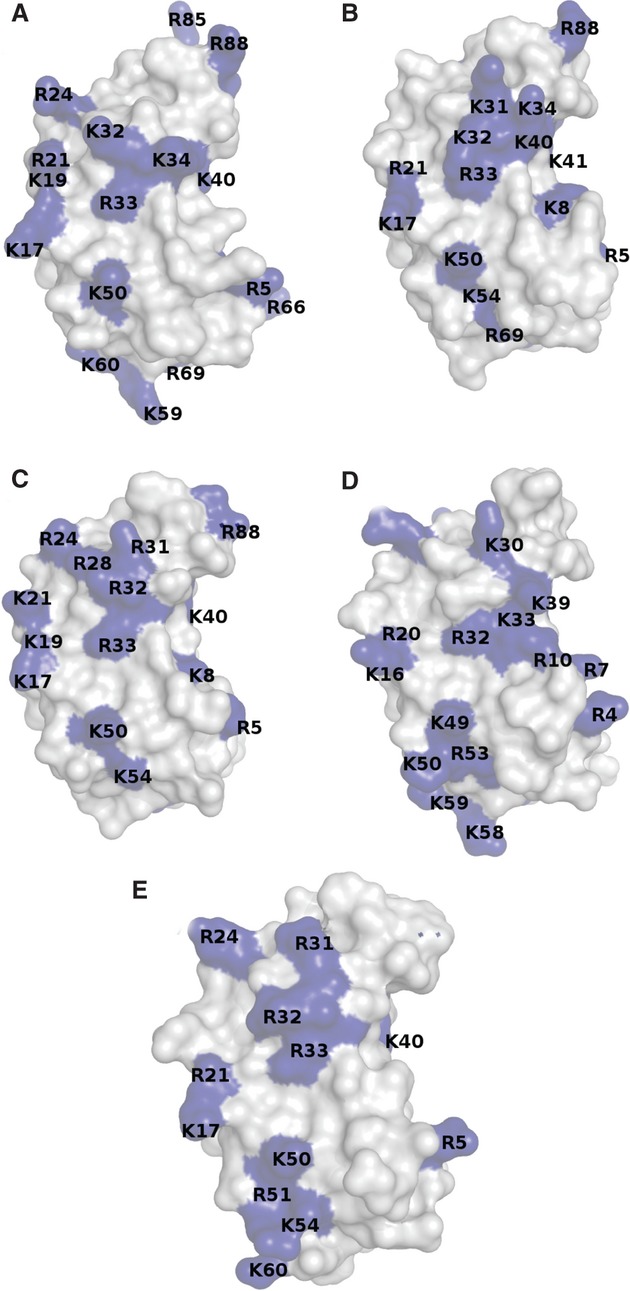
Positive charge around the NLS region. Surface representation of (A) mAng-2, (B) mAng-3, (C) mAng, (D) mAng-4 and (E) hAng. Positively-charged residues (lysine and arginine) in and around the vicinity of the NLS have been coloured blue.

Three consecutive positive charges (Arg31-Arg32-Arg33) comprise the NLS of hAng, of which Arg33 is the most crucial for nuclear targeting 52,53. Although the essential arginine is conserved (as Arg33 in mAng-2 and mAng-3), substitution of Arg31 for Val31 in mAng-2 alters the NLS chemical character. Arg31→Lys31 substitution in mAng-3 is subtle and preserves a surface charge distribution similar to that of hAng. Looking at the surface of mAng-2 and mAng-3, a series of positively-charged residues can be seen dotted around the NLS region (Fig. 7). The amino acid substitutions conjectured to provide a noncontiguous basic patch in mAng-4 35 could be hypothesized to do the same in mAng-2 and mAng-3.

**Fig 8:**
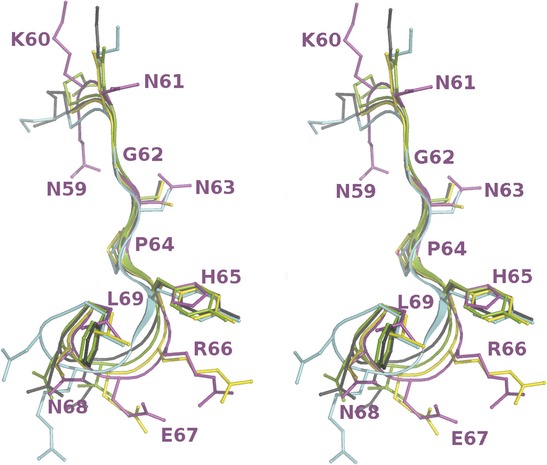
Stereoview of the cell-binding site. Schematic representations are shown in stereo for mAng-2 (greencyan) superposed with mAng-3 (splitpea green) and hAng (magenta). Residues are shown in stick form. Residues comprising the cell-binding region of hAng have been labelled in magenta. For the sequence of the putative cell-binding sites in mAng-2 and mAng-3, see Fig. 1.

The cell-binding site in hAng comprises residues 60–68 and Asn109 54,55. The sequence of the 60–68 segment is fairly well conserved in mAng but varies more in the other murine angiogenins (Fig. 1). In this region, the C^α^ traces of mAng-2 and mAng-3 (Fig. 8) deviate from the conformation observed for hAng 2, mAng 38 and mAng-4 35. Similar to that observed in mAng and mAng-4, this deviation is brought about by the replacement of the Glu-Asn sequence in hAng with a single Gly residue, upstream of the 60–68 loop region (a substitution found in all the murine angiogenins). Mutagenesis studies have highlighted Asn61 and Arg66 of hAng as amino acid residues crucial for angiogenic activity 54,55. The mAng-2 sequence has a Asn→Lys substitution similar to that in mAng-4 at position 60 (the counterpart of Asn61 in hAng and Lys59 in mAng-4). The critical Arg66 of hAng is replaced by a Gly residue (position 65) in both mAng-2 and mAng-3. Reduced steric constraints at this position on account of this glycine give the adjacent residues some freedom to deviate from the positions of their hAng counterparts, which is reflected in the rmsd of these residues. mAng-2 deviates the most from hAng with a C^α^ rms deviation of 2.35 Å (1.08 Å and 2.09 Å for mAng-3 and mAng-4, respectively). The position and conformation of residue Arg65 in mAng (equivalent to Arg66 of hAng) is similar to that of hAng (C^α^ displacement of 0.82 Å).

## Discussion

We have reported the crystal structures of two murine Ang paralogues. Even though these structures are broadly similar to the previously elucidated 3D structures of hAng, mAng and mAng-4, there are important albeit subtle differences amongst these proteins that have been highlighted in the course of the present study. These Ang structures have enabled us to carry out a more rigorous and authentic structure-based analysis than that presented by Codoñer *et al*. [36], which was based on 3D structural models for the different murine angiogenins. The most important difference between our analysis, based on our experimentally observed data, and theirs is the condoñer *et al* [36] suggest that a possible reason for the angiogenic incompetence of mAng-2 (unlike mAng) is a more effective blockage of its active site. In our structures, however, we observe that mAng-2 has a more open active site than hAng or mAng. This observation is consistent with the fact that mAng-2 is approximately three-fold more active against tRNA than is mAng (Fig. 2) Codoñer *et al*. [32] make a similar prediction for mAng-5 with regard to its lack of angiogenic activity, which can only be validated when the 3D structure is elucidated.

### Metal-ion binding

Divalent metal ions, such as Cu^2+^ and Zn^2+^, have been shown to inhibit the ribonucleolytic activity of hAng 56. Also, Cu^2+^ appears to prevent the formation of the Ang–RI complex and is considered to play an important a role in regulating its activity 57. Soncin *et al*. [58] showed that hAng directly interacts with copper and zinc ions. This interaction was found to result in the formation of Ang dimers and increased binding of the protein to lower affinity extracellular matrix binding sites. The dimers observed in the crystal lattice for mAng-2 and mAng-3 provide the first visual confirmation of the binding of an Ang to a divalent cation. It has been reported that RNase A binds copper at several sites (also involving a dimer of proteins) and its enzymatic activity is inhibited by copper and zinc, similar to angiogenin 56. The crystal structures of zinc-bound mAng-2 and mAng-3 provide some insight into the possible mode of inhibition of ribonucleolytic activity of the enzyme by these divalent cations. The present structures suggest that the tethering of the catalytically essential His113 (His114 in hAng) to a zinc ion at the active site could potentially prevent the participation of the active site His residue in general base catalysis. The mAng-2 structure also reveals Lys40 to be reoriented out of the active site pocket, making it ineffective as a catalytic residue. It is possible that copper binds hAng in a similar fashion. Perhaps other residues (e.g. Tyr14 as observed in the mAng-3 structure) are also recruited. It has been reported that iodination of hAng modifies the elution profile of the protein during copper-affinity chromatography 58. Because tyrosine residues are susceptible to iodination, Tyr14, which is in close proximity to His8 and His13, is a likely candidate. Interaction of mAng-2 His82 with zinc suggests that the corresponding loop region of hAng, which is known to interface with RI might also be affected by the binding of zinc ions. In hAng, this loop contains residues G85 and G86, which have been shown to interact with human RI (PDB code: 1A4Y) 23. Mutation studies have further shown that changes to these residues increase the angiogenic activity of the human protein and this increase can be attributed to a decrease in its affinity for RI 26. This provides a possible explanation regarding how copper might prevent the formation of the hAng–RI complex. Although the precise identity of the amino acid residues involved in binding copper are yet to be determined and it is not clear as to which mode of dimerization is adopted by hAng, the ions bound in the two structures reported in the present study give an insight into how divalent cations such as copper and zinc might interact with Ang and potentially modulate its function.

### Ribonuclease A activity and cell binding

**Fig 9:**
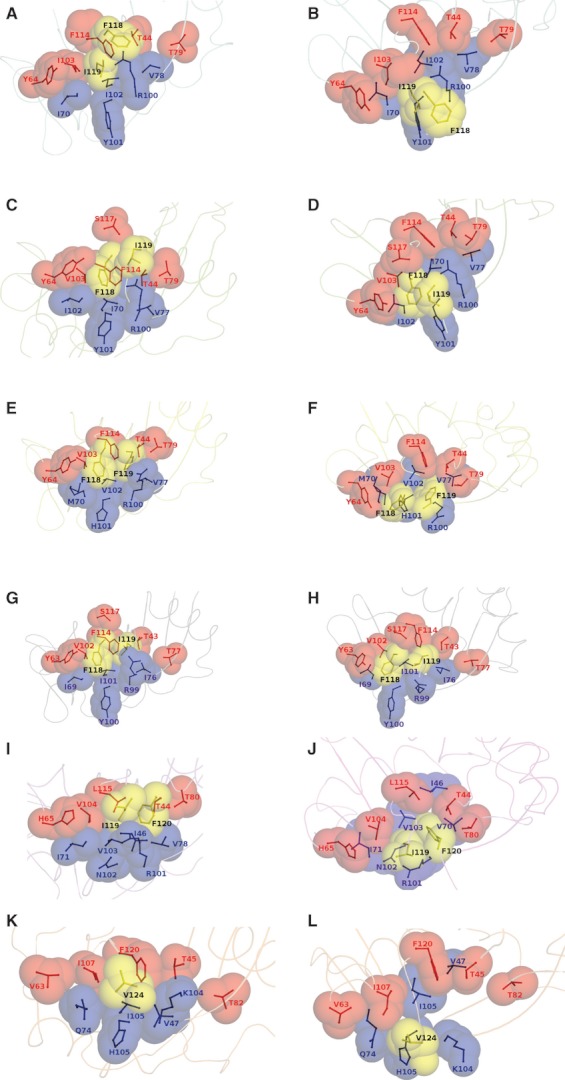
Hydrophobic packing of the C-terminus. (A, B) mAng-2, (C, D) mAng-3, (E, F) mAng, (G, H) mAng-4; (I, J) hAng; (K, L) RNase A. Hybrid representation in which the secondary structures are shown in schematic form and side-chains of the C-terminal hydrophobic residues (yellow), the pocket base (blue) and collar (red) are shown in both ball-and-stick form, as well as opaque space-filling spheres. (B), (D), (F), (H), (J) and (L) were obtained from (A), (C), (E), (G), (I) and (K), respectively, by a 90° rotation about the *x*-axis.

Despite structural similarity amongst the angiogenins, the enzymatic properties of these proteins exhibit some differences, as might be predicted from the sequence variations at amino acid positions that are involved in contributing to the catalytic properties of these proteins. All mAngs have a lower level of catalytic efficiency compared to hAng. The specific activity of mAng-3 is similar to that of mAng and mAng-4 35. mAng-2, on the other hand, appears to be able to cleave tRNA more efficiently (Fig. 2). Within the Ang family, it is considered that enhanced hydrophobic interactions between the C-terminus and the main body of the protein presents the enzyme with a higher energy barrier for substrate turnover and hence leads to suppression of catalytic activity. Studies whereby these hydrophobic interactions were weakened through mutagenesis or by the addition of methanol have shown that the catalytic prowess of hAng increases by three- to four-fold even though the substrate specificity remains unaffected 50,59. The C-terminal segment of the angiogenins, in general, is more nonpolar in nature than that of RNase A (Fig. 9). Hydrophobic interactions in the C-terminal region are potentiated in the murine angiogenins by the presence of Phe residues at positions 114 and 118 (113 and 117 for mAng-4), which may explain the lower catalytic efficiency of murine angiogenins compared to hAng, which possess Leu and Ile at the corresponding positions. Unfavourable positioning of the glutamate residue in mAngs (counterparts to hAng Gln117) causes obstruction of the pyrimidine-binding site. mAng (possessing Phe118/Phe119 and hence having a more hydrophobic C-terminus than other mAngs) and mAng-4 (possessing Phe117/Ile118) have a closed conformation similar to that of the human protein in which the side-chain of Phe119 (mAng)/Ile118 (mAng-4) also obstructs the substrate-binding site (Fig. 8). However, the side-chains of the equivalent pair of amino acids (Phe118/Ile119) do not block the active site in mAng-2 and mAng-3 (Fig. 6). This partial opening of the active site may explain why mAng-2 is enzymatically more efficient than is mAng and mAng-4; however, it does not explain why mAng-3 is not. Perhaps this can be rationalized by looking at the sequence of the different angiogenins at the N-terminal end (Fig. 1). Russo *et al*. 1996 provided kinetic evidence for the interaction of hAng Arg5 with a peripheral component of RNA substrates. They reported that hAng has functional phosphate binding sites on both the 5′ and 3′ side of the B_2_ subsite with greatest affinity for a 2′-phosphate. The Arg residue at this position is conserved across all angiogenins. However, amino acids on either side of this residue vary among the human and murine proteins. Although mAng-2 has the same residues before and after Arg5 (Ser4 and Tyr6), Ser4 is replaced by Tyr4 in mAng-3. It is possible that the larger side-chain of the Tyr residue perturbs the base binding site in mAng-3 causing a reduction in ribonucleolytic activity.

### Angiogenin–heparin interactions

Past studies have revealed that at least 12 saccharide units are required to inhibit the adhesion of hAng to HT-29 cells 60. The three contiguous arginines, Arg31/Arg32/Arg33, along with Arg70 were identified as important residues that mediate interactions with heparin to support cell adhesion to hAng. Harper and Vallee 1988 also showed that heparin prevents trypsin-mediated cleavage of hAng at Lys60, thereby suggesting that heparin might also bind to this region of hAng. The 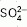 ions in the mAng-2 structure occupy sites between two mAng-2 molecules along a plane perpendicular to the dimer axis (Fig. 3), forming a crystallographic dimer. One of the 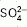 ions is shared between the two protomers. The shared 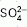 ion is located near the active site of the enzyme and ligates with P_1_ (Lys40) and B_1_ (Val42) subsite residues of both the mAng-2 protomers. Its interaction with Lys40 renders this residue unavailable to the catalytic core. Interaction with Asn67, Arg85, Ser86 and Arg92, observed at the other sulfate site, is almost exactly mirrored by the symmetry-related sulfate binding site at the opposite end of the dimer (Table 3). The side-chain orientation of Arg92 is maintained by its interaction with the main-chain carbonyl oxygen of Arg66. Although none of the sulfate ions in the mAng-2 structure bind the region equivalent to that comprising the Arg cluster in hAng, two of the sulfate ions do bind Arg66 and Asn67. These residues are located in the loop region equivalent to the segment of hAng (residues 60–68) that has been implicated in cell binding 54,55,62. Although the two sites are 37–38 Å apart, it is possible that loop 60–68 may form a subsite for heparin binding. The sulfate ions in mAng-2 are placed such that the distance between the shared anion and the one binding near the loop regions of 63–71 and 84–92 is ∼16 Å. A heparin molecule of five to six saccharides in length would be sufficient to bridge this distance, with the heparin chain extending from the tri-arginine cluster in the direction of the cell-binding site.

Light scattering measurements have determined a binding density of one hAng molecule for every five or six monosaccharide units 60. This is in contrast to the requirement of at least 12 saccharide units for inhibition of the capacity of hAng to support cell adhesion 60. In this regard, the fortuitous positioning of the sulfate ions in mAng-2 may provide some clues to the possible mode of heparin binding. The above findings suggest that hAng might form dimers to interact with the anionic heparin chain. The cell-binding site is critical for nuclear translocation of angiogenin and it may be that internalization of angiogenin is inhibited upon interaction with heparin as a result of the recruitment of residues from the cell-binding region. This can be explored further by elucidation of the structure of an angiogenin–heparin complex.

## Experimental procedures

### Protein expression and purification

Expression plasmids for both mAng-2 and mAng-3, each with a single copy of the coding sequence inserted into pET22b(+) (Novagen, Madison, WI, USA), were available from a previous study 63. The expression plasmids were used to transform BL21-CodonPlus(DE3)-RIL cells (Stratagene, La Jolla, CA, USA). Expression and purification of the recombinant proteins followed the method described by Holloway *et al*. [63]. Briefly, protein expression, induced by the addition of 1 mm isopropyl thio-β-d-galactoside at 37 °C, was directed to the insoluble fraction. These inclusion bodies were solubilized in 7 m guanidine hydrochloride and subjected to arginine-assisted refolding at pH 8.0. The refolded proteins were then purified by SP-Sepharose (cation-exchange) chromatography followed by C4 reversed-phase HPLC. The purified proteins were lyophilized and reconstituted in HPLC-grade water. The purity and the homogeneity of the proteins (> 98%) were monitored by SDS/PAGE. Protein concentration was determined by measuring UV absorbance and using an estimated ε_280_ 64 of 14 815 m^−1^·cm^−1^ for mAng-2 and 17 795 m^−1^·cm^−1^ for mAng-3. Both mAng-2 and mAng-3 were authenticated by ESI TOF-MS. The recorded molecular mass of each protein was accurate to within 1 Da of the predicted mass.

### Ribonucleolytic activity assay

tRNA cleavage assays were carried out as described by Shapiro *et al*. 65,66. Assays were conducted using 2 mg·mL^−1^ yeast tRNA (Sigma, St Louis, MO, USA), 0.1 mg·mL^−1^ BSA (Worthington Biochemical Corp., Freehold, NJ, USA) and six different concentrations (in triplicates) of each of the test proteins. hAng was used as reference sample. *A*_260_ was recorded as a measure of RNase activity. The readings were plotted against the protein concentration for each protein and the magnitude of activity was used in the calculation of relative activities. The activity of hAng was found to be in accordance with previous measurements.

### Crystallization and data collection

Crystals of mAng-2 and mAng-3 were grown at 16 °C by the hanging-drop vapor-diffusion method. mAng-2 crystallized within 3–4 weeks when crystallization buffer containing 25% poly(ethylene glycol) 550 monomethyl ether, 0.1 m 2-(N-morpholinoethanesulfonic acid, pH 6.5 and 0.01 m ZnSO_4_ was mixed with an equal volume of protein solution (16 mg·mL^−1^ in water). Diffraction data were collected from a single crystal, flash-frozen at 100 K on PX 9.6 at the Synchrotron Radiation Source (Daresbury, UK). Data were processed and scaled using hkl2000 67,68 in the tetragonal space group, *I*4_1_22. mAng-3 was crystallized by equilibrating the drops containing protein solution (25 mg·mL^−1^ in water) and reservoir solution (1:1) against crystallization buffer (25% poly(ethylene glycol) 3350, 20 mm sodium citrate, pH 4.6 and 2% glycerol). Diffraction data were collected from a single crystal, flash-frozen to 100 K on PX 14.2 at the Synchrotron Radiation Source. Data for mAng-3 were processed and scaled using hkl2000 67,68 in the monoclinic space group, *P*2_1_. Details of the data collection and processing for both mAng-2 and mAng-3 are given in Table 1.

### Structure determination and refinement

Initial phases for both the structures were obtained using amore 69, employing the coordinates of mAng (PDB code: 2BWK) 38 as the search model. The search model was tailored such that residues non-identical between mAng and mAng-2/mAng-3 were mutated to alanine. Data in the range 4–20 Å were used for both rotation and translation function searches. Clear solutions were obtained for both mAng-2 and mAng-3. Inspection of the resultant models with coot 70 revealed a convincing crystallographic packing environment without any stereochemical clashes.

Crystallographic refinement was carried out using refmac 71. A random set of reflections comprising 2.6% (mAng-2) and 5.2% (mAng-3) was excluded from the full data set for calculation of the cross-validatory free-*R* factor (*R*_free_) to monitor the refinement trend 72. After the initial round of refinement, the mutated residues in the model were replaced with the authentic mAng-2/mAng-3 residues on the basis of *mF*_o_ − *DF*_c_ electron density. Cycles of restrained refinement interspersed with electron density map calculations and manual model building using coot 70 progressively improved the structure. In the final stages of refinement, water molecules with peaks > 3σ in the *mF*_o_ − *DF*_c_ electron density map were incorporated into the structures. Zn^2+^ and 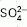 ions were also incorporated in both structures. Structure validation was carried out using molprobity 73 and the rcsb PDB Validation Suite (http://deposit.rcsb.org/validate/). Refinement statistics for the finalized structures of mAng-2 and mAng-3 are provided in Table 1. Figures were prepared using pymol (http://www.pymol.org).

## Abbreviations

ALS: amyotrophic lateral sclerosis
Ang: angiogenin
CSS: complexation significance score
hAng: human angiogenin
hRI: human ribonuclease inhibitor
mAng: murine angiogenin-1
mAng-2, -3, -4: murine angiogenin orthologues
NLS: nuclear localization sequence
PDB: Protein Data Bank
